# Sotn ureteroscope system with vacuum suctioning device for treating complicated steinstrasse: a case series

**DOI:** 10.3389/fsurg.2025.1520011

**Published:** 2025-05-07

**Authors:** Zhaolin Zhang, Shuiqing Xiao, Qingming Zeng, Linwei Liu, Tianpeng Xie, Xiaoning Wang

**Affiliations:** ^1^ Department of Urology, First Affiliated Hospital of Gannan Medical University, Ganzhou, Jiangxi, China; ^2^ First Clinical Medical College, Gannan Medical University, Ganzhou, Jiangxi, China

**Keywords:** steinstrasse, ureteral calculi, suction ureteral access sheath, ureteroscopic lithotripsy, stone free rate

## Abstract

**Objectives:**

To evaluate the safety and efficacy of sotn ureteroscope system with vacuum suctioning device for treating complicated steinstrasse.

**Materials and methods:**

The medical records of 22 patients with complicated steinstrasse who underwent ureteroscopic lithotripsy via sotn ureteroscope system with vacuum suctioning device from January 2020 to March 2023 were summarized retrospectively. Five patients had ipsilateral renal calculi. The 11.5/12.5 F sotn ureteral access sheath (sotn-UAS) was placed below the lowermost section of the steinstrasse assisted by a 7.5/9.8 F standard ureteroscope, and then a 4.0/6.0 F console ureteroscope with laser fiber replaced the standard ureteroscope and was used for pulverizing the steinstrasse. The vacuum suctioning device was connected to the sotn-UAS for suctioning fragments and dusts.

**Result:**

All procedures were successfully completed. The steinstrasse was free in 22 patients according to kidney-ureter-bladder radiography one day postoperatively. No intraoperative complications were observed. A total of four patients experienced postoperative complications, including one patient who experienced fever and was treated with antipyretics, one patient who experienced hematuria and was cured with hemostatic agents, and two patients who experienced urinary tract infection and needed only antibiotics. Five patients with ipsilateral renal calculi were treated with medical expulsive therapy or retrograde intrarenal surgery after steinstrasse surgery, and four of these patients achieved stone free status at the 3-month follow-up.

**Conclusion:**

The Sotn ureteroscope system with vacuum suctioning device is a feasible and safe treatment for complicated steinstrasse and provides satisfactory clinical outcomes.

## 1 Introduction

Urolithiasis is a global urological disease with a prevalence ranging from 1% to 13% in different geographical areas (1). Currently, extracorporeal shock wave lithotripsy (ESWL), and minimally invasive endoscopic surgical methods, including retrograde intrarenal surgery (RIRS) and percutaneous nephrolithotomy (PCNL), are the main therapeutic methods for treating urinary calculi.

Steinstrasse (SS) is an iatrogenic complication of the lithotripsy procedure, that occurs in 3%–7% of patients after ESWL (2) and in 1.0%-1.9% of patients after RIRS (3, 4). The major dilemma of SS is obstructive complications, such as renal colic, hematuria, fever, aggravation of hydronephrosis, and urinary tract infection or urosepsis (5). Most patients are symptom free and are often initially treated with conservative therapy initially including close surveillance and pharmacotherapy. For patients with symptoms or failure of conservative therapy, interventions such as repeat ESWL, ureteroscopy, PCNL, RIRS or combination therapy are recommended (6, 7). Complicated SS, defined as a total length of SS longer than 20 mm or the presence of more than 3 large stone fragments, presents a great challenge for treatment and lacks standardized treatment protocols (6).

The sotn ureteroscope system (sotn-URS) has both lithotripsy and suction features. A previous study demonstrated that sotn-URS is safe and effective for treating upper urinary calculi with satisfactory outcomes (8). However, the clinical application of sotn-URS in the treatment of SS is lacking. We present our initial experience with sotn-URS for treating complicated SS.

## 2 Materials and methods

### 2.1 Patients

The medical records of patients with complicated SS who underwent ureteroscopic lithotripsy via the sotn-URS in the First Affiliated Hospital of Gannan Medical University between January 2020 and March 2023 were retrospectively reviewed. Three patients whose sotn ureteral access sheath was unable to be inserted due to ureteral tortuosity or ureteral stenosis and who were converted to RIRS were excluded. A total of 22 patients with complicated SS were successfully treated with sotn-URS.

All patients underwent preoperative kidney-ureter-bladder graphy or non-contrast computed tomography. The stone size was defined as the longitudinal length of the SS on the basis of the radiological results. For discontinuous SS, the size was the sum of all segments. Urinalysis and urine culture were routinely examined, and the patients were treated with appropriate antibiotics preoperatively. Patients with positive urine cultures results received antibiotics according to sensitivity tests. Tamsulosin and traditional Chinese medicine were administered immediately after the SS was diagnosed.

The preoperative demographic characteristics, including gender, age, American Society of Anesthesiologists score, body mass index, SS side, cause of SS, midstream urine culture result, stone parameters, Coptcoat classification (9), and number of patients with ipsilateral renal calculi, were obtained according to medical records.

Ethical approval for the study was obtained from the Ethical Committee of the First Affiliated Hospital of Gannan Medical University (Number: 2023032701), and the study was conducted in accordance with the Declaration of Helsinki (as revised in 2013). Written informed consent was obtained from all participants included in the study.

### 2.2 Surgical techniques

**Figure 1:**
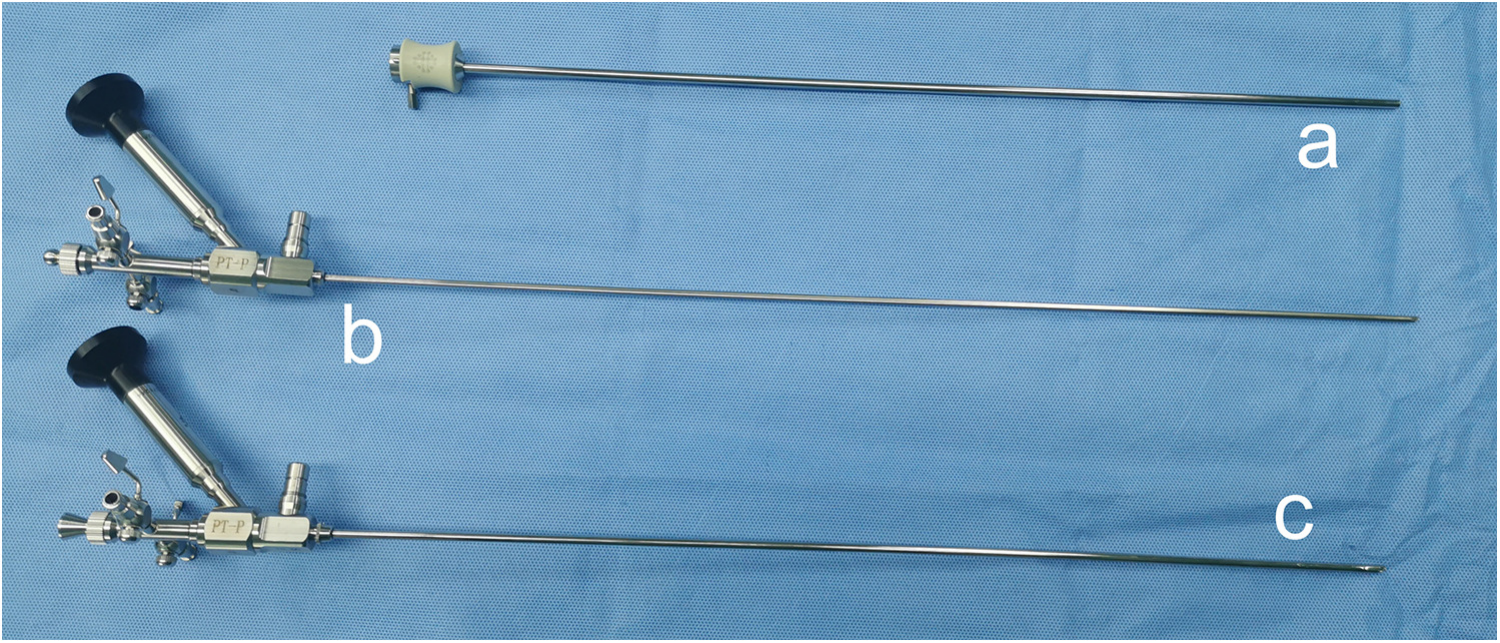
The suction ureteral access sheath **(a)**, console ureteroscope **(b)** and standard ureteroscope **(c)**.

The sotn-URS (ShuoTong Medical Company, Jiangmen, China) contains a suction ureteral access sheath (sotn-UAS) with an inner diameter of 11.5 F and an outer diameter of 12.5 F (Figure 1a), a console 4.0/6.0 F ureteroscope for lithotripsy (Figure 1b), a standard 7.5/9.8 F ureteroscope (Figure 1c) for facilitating the insertion of sotn-UAS, a T-shaped adapter, an irrigation device and a vacuum suction device.

**Figure 2:**
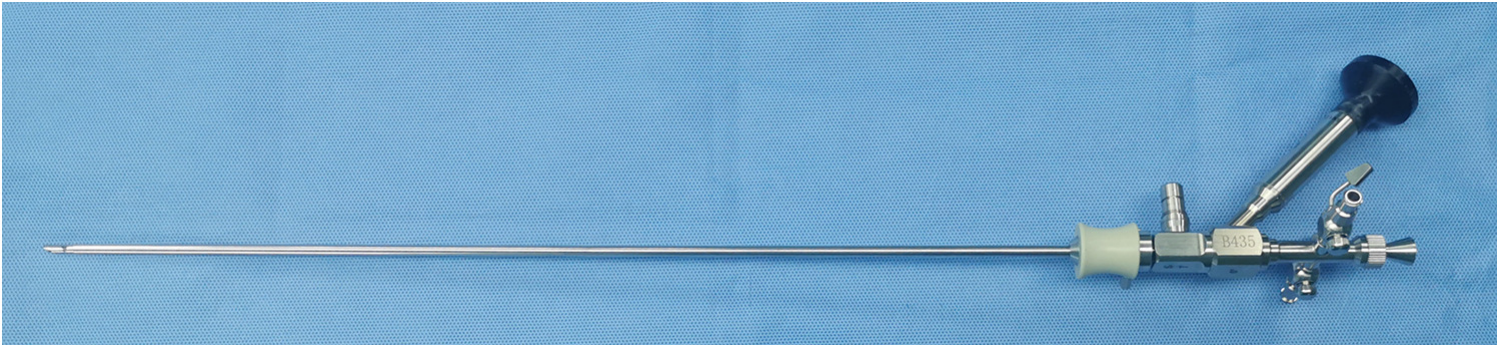
The suction ureteral access sheath combined with standard ureteroscope.

The lithotomy position was applied for all patients after satisfactory general anaesthesia. The preexisting double J tube was removed via the standard ureteroscope. Under the guidance of a hydrophilic 0.035-inch guide wire, the standard ureteroscope combined with the sotn-UAS (Figure 2) was inserted into the ureter and retrograded to the position of the lowermost SS fragment.

**Figure 3:**
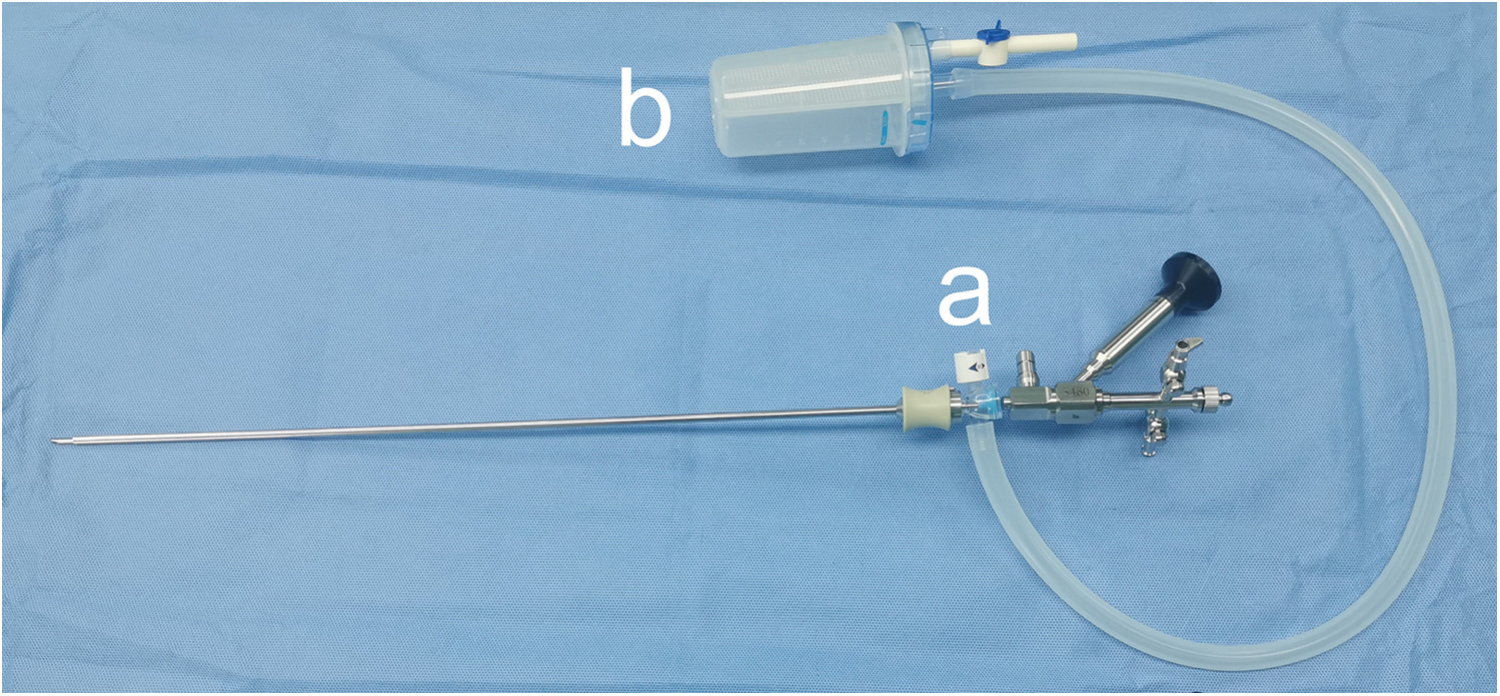
The suction ureteral access sheath, console ureteroscope, adapter **(a)** and collecting bottle **(b)**.

The sotn-UAS was advanced or retreated under the direct view of the ureteroscope. The standard ureteroscope was then replaced by a console ureteroscope. A horizontal port of the T-shaped adapter (Figure 3a) was connected to the sotn-UAS before ureteroscope insertion, and another horizontal port and a vertical port were connected to the ureteroscope and the collecting bottle (Figure 3b), respectively.

Another opening of the collection bottle was combined with a vacuum suction device. The vacuum parameter was set at −15 to −35 KPa and the vacuum effect could be manually adjusted by switching the adapter. The fluid was pumped through the ureteroscope into the ureter via the irrigation device, and the flow rate was set at 50–80 ml/min. A 365 μm laser fiber and a holmium laser were applied to pulverize calculi by interchangeably setting fragmentation and dusting modes. Higher energy (0.8–1.5 J) and lower frequency (5–20 Hz) modes were set for fragmentation, and the dusting mode was set to low energy setting (0.2–0.6 J) and high frequency (25–30 Hz). The dust and tiny fragments were suctioned out from the gap between the ureteroscope and sotn-UAS. For fragments larger than the gap but smaller than the inner diameter of the sotn-UAS, we retreated the ureteroscope slowly until beyond the T-shaped adapter, and the fragments were expelled following the ureteroscope. Stone forceps or baskets can be avoided. Intermittent retraction of the ureteroscope during the lithotripsy process was performed to avoid stone obstruction in the sotn-UAS. When ureteral tortuosity was encountered, the guide wire needed to be reinserted to facilitate ureteroscope advancement. The reverse Trendelenburg position was used for the proximal ureteral SS to reduce stone retropulsion. After confirming that the entire SS was cleared, a 5F or 7F double J stent was indwelled.

The operative time, hemoglobin loss, SS-free rate, postoperative hospitalization and complication rate were analysed. Intraoperative and postoperative complications were evaluated by the Satava classification (10) and Clavien‒Dindo classification (11), respectively. The kidney-ureter-bladder graphy was performed at 1 day postoperatively. SS-free was defined as no ureteral residual stone. The double J stent was removed regularly at 1–2 weeks after surgery. Patients with renal or ureteral residue fragments who required further therapy were treated 2–4 weeks after surgery. Routine follow-up was arranged for ultrasonography and/or kidney-ureter-bladder graphy and/or non-contrast computed tomography examination at 3 months after surgery. The final stone free rate was defined as no renal and no ureteral residual stones. Statistical analyses were conducted using SPSS 21.0 software (IBM, Armonk, NY, USA). Continuous data were recorded as the mean and standard deviation, and qualitative variables were expressed as percentages (%) or numbers (n).

## 3 Results

Twenty-two patients, including 12 males and 10 females, had a mean age of 52.82 ± 12.65 years. The mean SS size was 41.73 ± 15.77 mm, ranging from 22 to 87 mm. SS was classified according to the Coptcoat classication (9), and types I, II and III were observed in three, four and fifteen patients, respectively. Ten patients suffered SS after ESWL, and thirteen patients experienced SS secondary to RIRS. Five patients had ipsilateral renal stones. Percutaneous nephrostomy was performed in two patients due to fever and renal colic caused by SS; one patient had hematuria, four patients only experienced renal colic and were treated with analgesics before SS lithotripsy surgery, and the remaining patients had no symptoms. The demographic characteristics and baseline data are shown in Table 1.

**Table 1:** Demographic characteristics and baseline data of patients.

Parameters	Value
Total number, (*n*)	22
Age (years), mean ± SD	52.82 ± 12.65
Gender, *n* (%)	
Male/female	12 (54.5%)/10 (45.5%)
BMI (kg/m2), mean ± SD	24.03 ± 3.53
ASA score, *n* (%)	
I/II/III	5 (22.7%)/16 (72.7%)/1 (4.6%)
Steinstrasse side, *n* (%)	
Left/right	8 (36.4%)/14 (63.6%)
The cause of steinstrasse,	
ESWL	9 (40.9%)
RIRS	13 (59.1%)
Midstream urine culture, *n* (%)	
Positive/negative	4 (18.2%)/18 (81.2%)
Steinstrasse size (mm), mean ± SD	41.73 ± 15.77
Steinstrasse location, *n* (%)	
Proximal ureter	8 (36.4%)
Middle ureter	3 (13.6%)
Distal ureter	9 (41.0%)
Proximal and middle ureter	1 (4.5%)
Middle and distal ureter	1 (4.5%)
Combined with renal calculi	5 (22.7%)

SD, standard deviation; BMI, body mass index; ASA, American Society of Anesthesiologists; ESWL, extracorporeal shock wave lithotripsy; RIRS retrograde intrarenal surgery.

**Figure 4:**
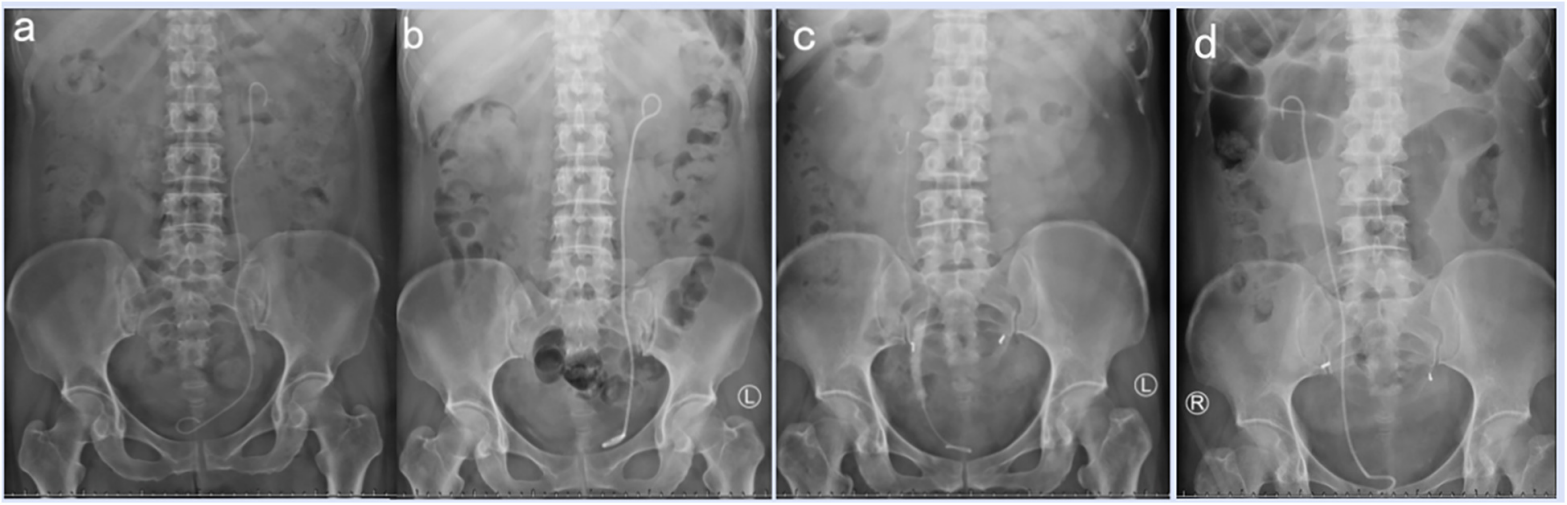
Steinstrasse cases. **(a)** preoperative KUB of patient 1, **(b)** postoperative KUB of patient 1, **(c)** preoperative KUB of patient 2, **(d)** postoperative KUB of patient 2.

The mean operation time was 54.32 ± 10.04 min, and the mean postoperative hospital stay was 2.86 ± 1.39 days (range, 1-7 days). The SS-free rate was 100% one day after surgery, and no stone retropulsion was observed. No intraoperative complications, including hemorrhage, ureteral perforation or tear, were encountered. A total of four patients experienced postoperative complications, including one patient who experienced fever and was treated with antipyretic agents, one patient who had hematuria and was relieved with hemostatic agents, and two patients who suffered urinary tract infection and were cured with antibiotics only. Some representative steinstrasse cases are presented in Figure 4.

For five patients who had ipsilateral renal stones, RIRS or PCNL was not performed simultaneously during the SS procedure. All patients were treated with medical expulsive therapy (tamsulosin and traditional Chinese medicine) after treating SS. Two patients expelled all the stones, and one patient still had residual stones in the low renal pole but refused further therapy. The remaining two patients underwent RIRS and were stone free at the 3-month follow up. The final stone free rate at 3 months after the SS lithotripsy procedure was 95.5% (21/22). No intraoperative or postoperative complications were observed in the RIRS procedures. During the follow-up period, no ureteral stricture was observed in any of the patients. The outcomes of these patients are shown in Table 2.

**Table 2:** Clinical outcomes of patients.

Parameters	Value
Total number, (n)	22
Operative time (min), mean ± SD	54.32 ± 10.04
Hemoglobin loss (g/dl), mean ± SD	−0.44 ± 0.70
Postoperative hospitalization (days), mean ± SD	2.86 ± 1.39
SS free rate at postoperative day 1, *n* (%)	22 (100.0%)
Total complications, Clavien grade classification, *n* (%)	4 (18.2%)
Fever (>38°C) (G I)	1 (4.5%)
Hematuria (G I)	1 (4.5%)
Urosepsis only need additional antibiotics (G II)	2 (9.1%)
Stone composition, *n* (%)	
Calcium oxalate	13 (59.2%)
Calcium phosphate	7 (31.8%)
Uric acid	1 (4.5%)
Struvite	1 (4.5%)

SD, standard deviation; G, grade.

## 4 Discussion

SS is an iatrogenic disorder and is secondary to mainly ESWL; some cases are due to RIRS (12) and PCNL (6). In our study, thirteen patients (59.1%) received SS after RIRS, and the remaining nine patients suffered SS secondary to ESWL. The main reason was that RIRS was increasingly favour by urologists and patients due to its high stone free rate and low complication rate, and patients preferred this minimally invasive transureteral orifice endoscopic surgery to PCNL, including patients with a large stone burden. Most ESWL procedures and simple SS complications were performed in secondary hospitals, and complicated SS patients needed to be referred to tertiary hospitals, such as our hospital, for further therapy.

Although most SS are cleared spontaneously, 6% of patients still require intervention (7). Conservative therapies including patient waiting, movement, and medical expulsive therapy, are initially recommended to accelerate the spontaneous passage of the SS. Adjunctive tamsulosin therapy could facilitate the expulsion of SS (13, 14) and reduce the need for further interventions (15). Chinese traditional medicine was also applied in our study. For patients who fail conservative therapy or who have persistent symptoms or the occurrence of obstructive complications, further treatment modalities including double J stent insertion, percutaneous tube, repearted ESWL, URL (16), PCNL or open surgery are advised (17).

Ureteroscopic lithotripsy (URL) is a safe and effective treatment for SS (6, 16). Feng et al. presented their experience with URL in treating SS (16). In total, 19 patients successfully received URL and were stone free at 1 month after surgery; one patient underwent postoperative ESWL due to stone retropulsion, and the other patient was converted to open surgery because of a distorted ureter. Wang et al. reported the application of a 12/14F vacuum-assisted ureteral access sheath (vaUAS) combined with a 7/8.4 Fr ureteroscope in complex SS; the immediate SS-free rate was 100%, and no stone retropulsion was observed (6). In our study, we used sotn-UAS to reduce stone retropulsion, and no patents experienced stone retropulsion or upwards migration. The sotn-UAS combined with the vacuum suction system could provide negative pressure effect, which would drive stone fragments to the opening of sotn-UAS and prevent stone retropulsion. The irrigation fluid entered the ureter through the ureteroscope and was suctioned out via the sotn-UAS, which created a flush/reflex cycle to reduce upwards fluid flow and accumulation. Thus, the proximal segment of the SS cannot be flushed retrogradely. The use of a stone occluder was avoided during our surgical procedures, which could reduce the total cost and simplify surgical manipulation. For the proximal ureteral SS, we changed to the reverse Trendelenburg position to decrease the possibility of stone retropulsion.

The SS-free rate at 1day post-surgery in our study was 100% (22/22), which was comparable with that (100%, 35/35) reported in Wang's study (6) and higher than that reported in traditional URL studies (16, 18). Feng et al. reported an SS-free rate of 90.48% at 1 month after surgery, but the immediate rate after surgery was unknown (16). Rabbani et al. reported the outcomes of transureteral lithotripsy in the treatment of 24 patients with SS; 58.3% achieved stone-free status, 25% achieved partial success, and the remaining four patients were converted to open ureterolithotomy (18). These differences attributed mainly to the different therapeutic mechanisms of stone extraction. In traditional URL procedures, two stone extraction methods were used: first, the calculi were pulverized into small debris and were removed via a stone basket or forceps; second, the stones were crushed into powder or tiny dusts and then passed spontaneously after surgery (19). However, unlike the above methods, active suction removal of fragments and dust was the main technique of sotn-URS, which could clear almost all stone fragments immediately and decrease possibility of stone retention. Tiny dust passed through the gap between the ureteroscope and the sotn-UAS, and lager fragments were suctioned out as the ureteroscope retreated. This removal style reduced or prevented the use of stone baskets or forceps, which could decrease the cost and reduce ureteral injury during the use of removal devices. A larger gap could improve stone removal efficiency and increase the stone free rate (20). Compared with the gap between the 7/8.5 F ureteroscope and 12/14 F vaUAS in Wang's study, our gap between 4/6 F ureteroscope and 11.5/12.5 F sotn-UAS was larger, but the SS-free rates in the two groups were comparable, which may be related to different patients and SS characteristics.

Our mean total operative time (54.32 ± 10.04 min) was shorter than that of Feng's study (60 min) (16), but the lithotripsy time was not recorded in our respective study. Wang et al. calculated the average lithotripsy time (33.7 min), but the total operative duration was not reported (6). In our study, the fragments and dust passed out under the action of negative pressure suction instead of stone baskets or forceps, which could decrease the re-entry times of extraction devices and shorten the total surgical time. For tiny fragments, the holmium laser and fiber were not applied. The turbulent vortex created by irrigation fluid could wash out the dust and fragments in a timely manner and continually maintain a clear surgical view, which can avoid missing stones and improve the efficiency of stone removal.

The overall complication rate was 18.2% (4/22), which was consistent with the results of Wang's study; however, the complication rate was not reported by traditional URL studies (16, 18). Fever and urinary tract infection were observed in our study, but no urosepsis or septic shock occurred, which may be due to the effect of sotn-UAS. In RIRS, the application of a ureteral access sheath reduces intrapelvic pressure and leads to a lower risk of infectious complication (21). Like UAS in RIRS, sotn-UAS in the ureter would theoretically reduce the intraureter pressure, facilitate drainage of irrigation fluids and decrease infectious complications. Infectious substances including bacteria released from stone fragments, bacterial endotoxins, suppurative flocs and abscess pus, were suctioned out in a timely manner, which could reduce the amounts of infectious substances absorbed into the bloodstream. Although the vaUAS was also used for treating SS in Wang's study, urosepsis was observed in one patient, and this difference may be related to the gap size between the ureteroscope and the vaUAS; however, few studies have focused on the relationship between the gap and infectious complications (20, 22), and further studies are needed to verify this hypothesis.

In addition, the insertion of sotn-UAS facilitated ureteroscope access and reduced injury to the ureter (21). In traditional URL, ureteroscope and stone extraction devices must re-enter and re-exist to remove stone fragments, which may increase the risk of ureteral injury and result in the absence of the ureteral orifice. The stone fragments and dust were suctioned into the sotn-UAS and lithotripsy procedures were limited the sotn-UAS, which could reduce ureter injury resulting from laser energy and stone disintegration. Traxer and Thomas reported that the percentage of patients with high grade ureteral wall injuries related to the ureteral access sheath was up to 13.4% (23). In our study, no ureteral injury was observed, predominantly because the insertion and removal of sotn-UAS were performed under direct vision of the ureteroscope.

In addition to the application of the sotn-UAS in the treatment of SS, the advantages of high stone clearance rates and low complication rates in the management of upper urinary tract stones have been demonstrated (24, 25).

Our current study has several limitations. This was a retrospective study without a control group and the number of patients was small. Selective bias cannot be avoided. Another limitation of our study was that the stone-free rate was not defined on basis of CT scans. A prospective randomized controlled study with a large number of cases is suggested, and pre-operative and postoperative urinary computed tomography are needed.

## 5 Conclusion

The Sotn ureteroscope system with vacuum suctioning device is feasible and safe for treating complicated steinstrasse and provides satisfactory clinical outcomes.

## Data Availability

The original contributions presented in the study are included in the article/Supplementary Material, further inquiries can be directed to the corresponding author.
